# Informational laws of genome structures

**DOI:** 10.1038/srep28840

**Published:** 2016-06-29

**Authors:** Vincenzo Bonnici, Vincenzo Manca

**Affiliations:** 1 University of Verona, Department of Computer Science, University of Verona, Verona 37134, Italy; 2 Center for BioMedical Computing, University of Verona, Verona, 37134, Italy

## Abstract

In recent years, the analysis of genomes by means of strings of length *k* occurring in the genomes, called *k*-mers, has provided important insights into the basic mechanisms and design principles of genome structures. In the present study, we focus on the proper choice of the value of *k* for applying information theoretic concepts that express intrinsic aspects of genomes. The value *k* = lg_2_(*n*), where *n* is the genome length, is determined to be the best choice in the definition of some genomic informational indexes that are studied and computed for seventy genomes. These indexes, which are based on information entropies and on suitable comparisons with random genomes, suggest five informational laws, to which all of the considered genomes obey. Moreover, an informational genome complexity measure is proposed, which is a generalized logistic map that balances *entropic* and *anti-entropic* components of genomes and is related to their evolutionary dynamics. Finally, applications to computational synthetic biology are briefly outlined.

The study of complexity in Biology is an old topic that often reemerges in theoretical biological investigations^1^,^2^,^3^. The study of complexity has very important implications for any deep understanding of the informational organization that life chooses in the different species to realize their specific biological functionalities. Entropy is a fundamental scientific concept that is naturally related to complexity and was the basis of statistical physics founded by Ludwig Boltzmann and the essence of his famous H theorem, which related the arrow of time to Boltzmann’s equation, where entropy is expressed in terms of mechanical microstates^4^. Essentially, the same function was the basis of the information theory founded by Claude Shannon in 1948^5^, where entropy is defined on information sources, that is, probability distributions over finite sets of elements (symbols, words or signals). A genome is essentially a text; if read at pieces of length *k* (called *k*-mers), a genome becomes an information source. Therefore genomic *k*-entropies can be easily defined, and the concepts and results of information theory can be applied^6^,^7^,^8^,^9^,^10^.

In recent years, many studies have approached the investigation of DNA strings and genomes by means of algorithms, information theory and formal languages^11^,^12^,^13^,^14^,^15^,^16^,^17^,^18^,^19^,^20^,^21^,^22^, and methods were developed for investigating whole genome structures. In particular, dictionaries of words occurring in genomes, distributions defined over genomes, and concepts related to word occurrences and frequencies have been very useful and seem to characterize important genomic features relevant in biological contexts^23^,^24^,^25^,^26^,^27^,^28^,^29^,^30^. Dictionaries are, in essence, finite formal languages. In genome analyses based on dictionaries, concepts from formal language theory, probability, and information theory are naturally combined by providing new perspectives in the investigation of genomes, which may disclose the internal logics of their structures.

The set of all *k*-mers, occurring in a given genome is a particular dictionary. A point that is crucial in genome analyses based on *k*-mers is the value of *k* that is more adequate for specific investigations. This issue becomes extremely evident when computing the entropy of a genome. We prove that preferential lengths exist for computing entropies, and in correspondence with these lengths, some informational indexes can be defined that exhibit “informational laws” and characterize an informational structure of genomes. As we have already noticed, there is a long tradition in investigating genomes by using *k*-mers. However, comparing genomes of different lengths, by using the same value of *k* (usually less than 12) may result in the loss, in some cases, of important regularities. In fact, the genomic laws that we discover emerge when the values of *k* are suitably defined from the logarithmic length of the genomes.

When genomic complexity is considered, it is very soon clear that it cannot be easily measured by parameters such as genome length, number of genes, CG-content, basic repeatability indexes, or their combinations. Therefore, we follow an information theoretic line of investigation based on k-mer dictionaries and entropies^16^,^26^,^27^,^31^,^32^,^33^, which is aimed at defining and computing informational indexes for a representative set of genomes. This task is not trivial when genome sizes increase, so a specific software package is used to this end^31^. Moreover, an aspect that is missing in classical Shannon’s conceptual apparatus is relevant in our approach: random strings and pseudo-random generation algorithms, which now can be easily produced and analyzed^34^. In fact, it is natural to assume that the complexity of a genome increases with its “distance” from randomness^35^,^36^, as identified by means of a suitable comparison between the genome under investigation and random genomes of the same length. This idea alone provides important clues about the correct *k*-mer length to consider in our genome analyses, because theoretical and experimental analyses show that random genomes reach their entropic maxima for *k*-mers of length lg_2_(*n*), where *n* is the genome length. No assumption on the distribution of probability of *k*-mers is assumed or inferred (as in Markov Models-based approaches); rather, data processing is developed on the basis of the empirical distributions of *k*-mers computed over the investigated genomes.

To this end, two basic indexes are introduced, which we call *entropic* and *anti-entropic components*. These indexes, and other related indexes, are computed over the chosen seventy genomes, ranging from prokaryotes to primates. The obtained values suggest some laws of genome structure. These laws hold in all of the investigated genomes and motivate the definition of the genomic complexity measure *BB* proposed in the paper. This measure depends on the entire structure of a genome and considers, together, the components of genomes (e. g., repeats, CpG, long range correlations, surely affecting entropies) without considering them separately. Moreover, as demonstrated below, *BB* is related to phylogeny but does not coincide with phylogenetic ordering. Certainly, primate genomes are usually more complex than, say, bacterial or insect genomes, but the situation is surely more critical because evolution is always active and a bacterium that we sequence today is not a type of bacteria that firstly arose in the tree of life. For this reason, genomes that are phylogenetically older can cumulate, even along different paths, “distances” from their corresponding random genomes comparable with those gained by “more evolved” genomes.

## Results

The results presented in this paper are based on comparing real genomes with random genomes of the same length. As we show, any genome 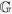 of length *n* defines a partition of lg_4_(*n*) in two addends 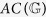 and 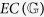 such that 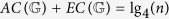.

### The fundamental informational components of genomes

We denote by 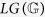 the value 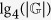. Of course, 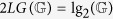. We call 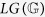 the logarithmic length of 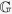 and 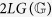 the *double logarithmic length* of 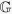. When no possible confusion can arise, we avoid explicitly indicating 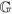, so we write in short *LG*, and consequently we denote the entropy 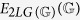 over the 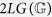-mers of 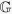 by 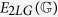 (analogous abbreviations are also adopted for other indexes). We also refer to the interval 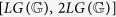 as the *critical entropic interval*. In the following, when 2*LG* is not integer, 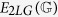 denotes the linear interpolation between 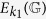 and 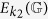, where *k*_1_, *k*_2_ are the smallest integers such that *k*_1_ < 2*LG* < *k*_2_. In the case of the human genome, 2*LG* is between 31 and 32; in the genomes considered in this paper (from microbes to primates), it ranges between 16 and 36.

We prove, by using well-known results of information theory, that the values *LG* and 2*LG* have the following properties (see section *Methods*):

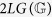
 is an upper bound to the values that entropy can reach over the genomes with the same length of 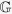;
- if *k* belongs to the critical interval 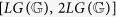, and 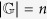, then entropies *E*_*k*_, for *k* ≤ *n*, reach, on suitable genomes, the best approximations to 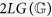 with an error close to zero, which is inferior to 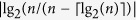, being 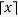 the closest integer greater than *x*.
- entropy 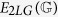 reaches its maximum in random genomes of length 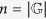. This result follows from the fact that in random genomes of length *n* all lg_2_(*n*)-mers are hapaxes, that is, they occur once in the whole genome^37^.

In conclusion, the maximum of 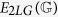 is almost equal to 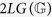, and this maximum is reached by random genomes of length 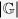. It was realized that for all of the investigated genomes the following inequality immediately holds:


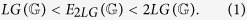


Therefore, we know that 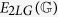 belongs to the (open) real interval of bounds 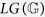 and 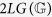. Then, we can define the following values 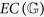 and 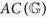, which we call *Entropic Component* and *anti-entropic Component* of 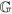, respectively:


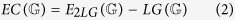



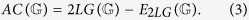


Summing Equations (2) and (3), we obtain 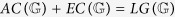. The value 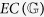 corresponds to the gap between the double logarithmic entropy 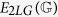 and the logarithmic length 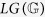, which is always positive according to the equations above. Moreover, 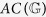 is the gap between the double logarithmic length 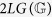 and the entropy 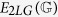, which is positive because 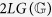 is an upper bound to the entropies in the critical entropic interval. The term “anti-entropic” stresses an important difference with the analogous concept of *neghentropy*, which is frequently used to denote the other side of the order/disorder dichotomy associated with entropy (and its time arrow)^38^,^39^,^40^,^41^. In fact, in *anti-entropy*, no change of sign is involved, but a difference from an upper bound of the entropy is instead considered.

### Informational genomic laws

Let us define 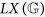, called *lexical index*, as the ratio:


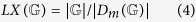


The numerator is essentially the number of words of length 2*LG* occurring in random genomes, which as we already noticed are all hapaxes, and therefore, coincides with the number of possible occurrences of 2*LG*-mers in 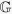. The denominator is the number of words of length 2*LG* occurring in 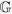. This ratio is related to the degree of order that 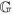 gains with respect to random genomes. In fact, in a random genome *R*, we have *LX*(*R*) = 1; therefore, in a real genome 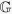, 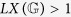. The lexical index is smaller than the ratio 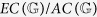 but is greater than 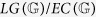. Moreover, by dividing and multiplying *LX* by 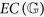 and 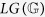, it is possible to obtain lower and upper bounds to 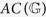. The value 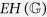, given by 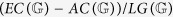, corresponds to the eccentricity of an ellipse associated with 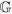 (see Supplementary Information, Sup. Fig. 3). The product of 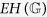 with 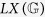 differs by 1 less than 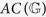. In conclusion, the following laws hold for all seventy investigated genomes:


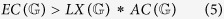



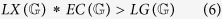



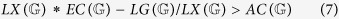



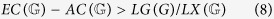



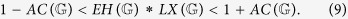


### Biobit: a measure of genomic complexity

As we already noticed, *AC* is an index measuring the informational distance between genomes and random genomes with the same length. This means that the more biological functions a genome 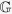 has acquired, the further the genome is from randomness. However, if we directly identify the complexity of 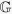 with 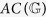, we obtain some biologically inconsistent results. For example, *Zea mays* has an *LG* value of 15.4701 but an *AC* value of 3.6678 (primates have *AC* less than 1). These types of anomalies suggested to us that *AC* is surely related to the biological complexity of a genome, but this complexity is not a linear function of *AC* because also the *EC* component also has to be considered in a more comprehensive definition of complexity. Our search focused on a function that combines *AC* with *EH*, which is strictly related to *EC*. If *x* briefly denotes the *anti-entropic fraction AF* = *AC*/*LG*, it is easy to verify that because *EC* = *LG* − *AC*, then *EH* = (*EC* − *AC*)/*LG* = (1 − 2*x*); therefore, the product *AC* * *EH* can be represented by:


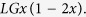


This function (after a simple change of variables) is a type of logistic map *ax*(1 − *x*), with *a* constant, and *x* variable ranging in [0, 1], which is very important in population dynamics.

If we generalize *x*(1 − 2*x*) in the class of functions *x*^*γ*^(1 − 2*x*)^*δ*^, with *γ* and *δ* positive rationals weighting the two factors, then we discover that these functions have maxima for values approaching to zero when *γ* ≤ 1 decreases and *δ* increases. Therefore, because *AC* is supposed to have a predominant role in the complexity measure, we define *BB*_*γ*,*δ*_ as *BB*_*γ*,*δ*_ = *x*^*γ*^(1 − 2*x*)^*δ*^ by choosing the values of the exponents in such a way that maxima of *BB*_*γ*,*δ*_ fall close to the values that the anti-entropic fraction *AF* assumes for the most part in genomes with high values of *AC* (almost all of them have medium horizontal eccentricity; see Supplementary Information, Sup. Table 2). No genome on our list reaches the maximum of the chosen function because their *AF* value is always smaller (suboptimal genomes) or greater (super-optimal genomes) than the value where the maximum is reached.

In conclusion, we conjecture that the genomic complexity is a non-linear function of *AC* having the form (apart from a multiplicative constant):


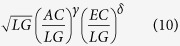


In particular, the following definition, which is an instance of (10), was supposed to be the most appropriate (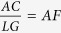 and 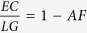):


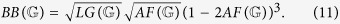


**Figure 1:**
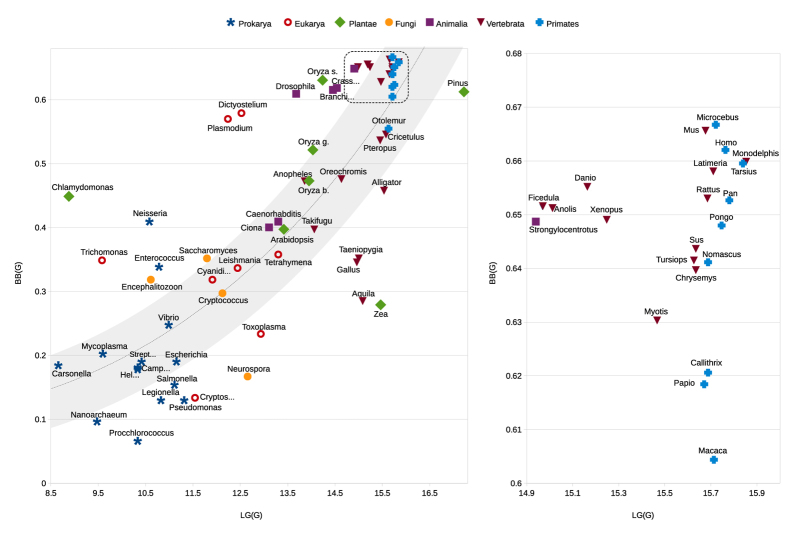
The left side of the figure shows the 70 analyzed genomes plotted on a Cartesian plane with their logarithmic length 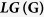 as the abscissa and their biobit value 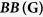 as the ordinate. An enlargement of the top-right region, which is highlighted with a dashed line, is shown on the right side of the image.

**Figure 2:**
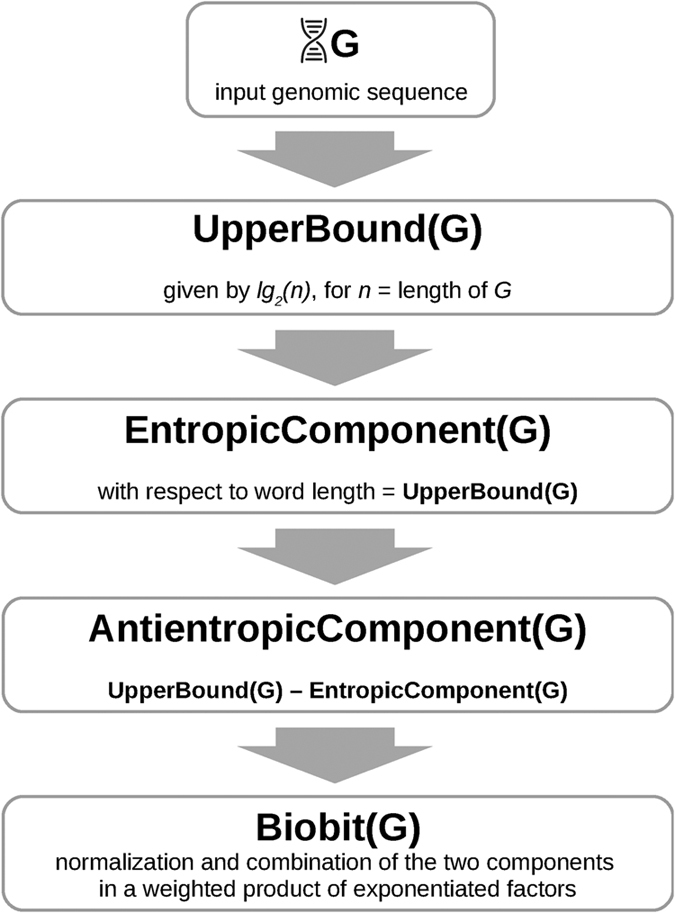
A flowchart of the computational steps involved in calculating 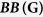. Given an input genome 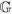, an upper bound of maximum entropy is calculated, its value equals 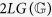, and the value also defines the appropriate word length. Then the entropic and anti-entropic components are computed as, respectively, 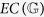 and 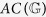 and are successively normalized and combined by a weighted product into 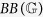.

In Fig. 1, the biobit values, together with the other described informational indexes, of the seventy genomes are visualized in a diagram. In Fig. 2 a flowchart is given that, in general terms, expresses the main stages for computing the *BB* measure of a given genome.

A further law could be associated with the biobit index, according to which genomes *evolve* by increasing the value of the *BB* function. This means that an ordering, denoted by 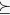 (a reflexive, antisymmetric, and transitive relation), can be defined such that:


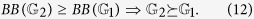


**Figure 3:**
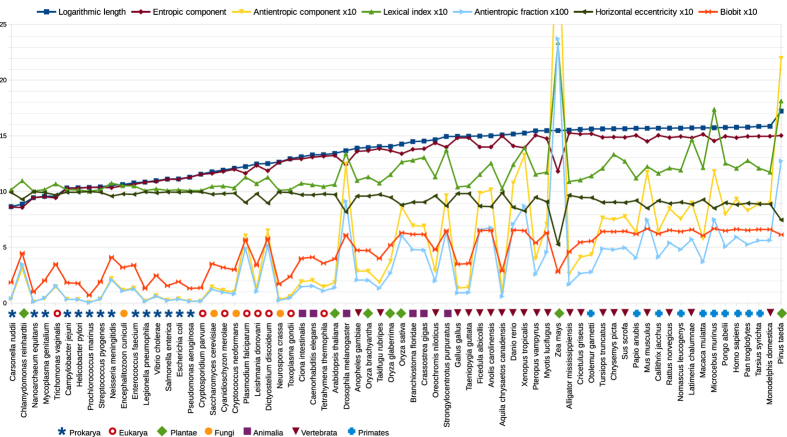
A chart of the main informational indexes. Some measures have been rescaled, by applying a factor of ten (×10) or one hundred (×100) to their value, to obtain a comprehensive overview. Species are arranged on the horizontal axis according to their genome length (increasing from left to right).

Table 1 reports the main informational indexes based on the two entropic components of the logarithmic length of genomes. Figure 3 depicts graphically the values of these informational indexes for all of the investigated genomes (see Supplementary Information, Sup. Table 4, for the exact numerical values). The lengths of genomes are naturally linearly ordered, thus allowing us to arrange them along the *x*-axis. Apart from the *EC* curve, which is quite coincident with *LG*, the other indexes presents peaks that correspond to the genomes differing only slightly in lengths but differing greatly in other indexes.

It is interesting that, in essence, biological evolution is anti-entropic because the *AC* component, representing the tendency toward order, increases with the increase of biological functionalities, under the constraint of keeping the ratio *AC*/*EC* under a threshold, as expressed by the factor (1 − 2*x*)^3^ of *BB*.

A 3D-visualization of our seventy genomes, by means of the *AC*, *LX*, *BB* informational indexes (see Supplementary Information, Sup. Fig. 2), reveals that genomic complexity does not coincide with classical phylogenetic classifications, as argued in the next section.

## Discussion

We think that our informational indexes, and the laws relating them, confirm a very simple and general intuition. If life is information represented and elaborated by means of (organic) molecules, then the laws of information necessarily have to reveal the deep logic of genome structures.

The laws presented in the previous section represent universal aspects of genome structure and may rarely hold for strings of the same lengths that are not genomes. Therefore, the genomic complexity measure BB, obtained by means of informational indexes, is not a mathematical trick but must to be related to the way genomes are organized and to the way in which the genomes were generated. Figure 2 shows the values of BB along the 70 investigated genomes, and it is clear that BB is related to the evolutionary positions of organisms. However, our approach has an important biological implication in clarifying the difference between phylogenesis and genomic complexity, which are related but different concepts. In fact, several cases have been found (see Fig. 2 and Sup. Fig. 2 in Supplementary Information) where organisms that are phylogenetically more primitive than others, for example bacteria, have biobit values greater than those of “more evolved” organisms. The reason could be the following. A bacterium that we sequence today is an evolutionary product of some primitive bacterium. Even if we do not know the path from the bacterium’s (possibly unknown) ancestor to the bacterium, its complexity along this path grew over time because its evolutionary age is the same as H. sapiens (even along different branches). The genomic complexity of 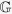 is, in a sense, a measure of the relevant steps from random genomes to 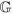. Surely, these steps reflect the evolutionary pressure and the biological interactions and competitions among species. However, if we forget this perspective, we lose an important aspect of evolutionary dynamics. This is why complexity-driven classifications that completely agree with phylogenesis are almost impossible. For example, we found that bacteria associated with human diseases have BB values significantly higher than others phylogenetically comparable to them. The BB measure is a sort of absolute distance from random, whereas phylogenesis concerns similarity or dissimilarity between species. Therefore, a very natural question arises, which suggests the development of the presented theory. Can entropic divergences (Kullback-Leibler divergence or similar concepts) be applied to phylogenetic analysis of genomes by means of “common words” and their probability distributions in the compared genomes? Finally, what is the applicability of our indexes in the identification of informational features that are relevant in specific pathological genetic disorders? Of course, these questions deserve specific investigations; however, our informational indexes with the related laws, and computational tools, provide a framework on which these informational analyses may be fruitfully set. We argue that it is almost impossible that functional changes do not correspond to precise informational alterations in the relationships expressed by the genomic laws. The challenge is in discovering the specific keys of these correspondences.

We developed some computational experiments showing a direct applicability of informational indexes and related genomic laws to the emergent field of synthetic biology. In fact, recent experiments on minimal bacteria^42^ are based on the search for genome sequences obtained by manipulating and reducing some real genomes. It has been proved that after removing some parts of the *M*. *mycoides* genome, the resulting organism, JCVI-syn3.0 (531 kilobase pairs, 473 genes), is able to survive and has a genome smaller than that of any autonomously replicating cell found in nature (very close to *M*. *genitalium*). Of course, in this manner a better understanding of biological basic functions is gained, which directly relates with the investigated genome (removing essential portions results in life disruption). On the basis of this principle, we considered *M*. *genitalium* and removed some portions of its genome through a greedy exploration of the huge space of possibilities. At every step of our genome modifications (of many different types), we checked the validity of our genomic laws. We found that, after removing portions of the genome, some of our laws do not hold in the resulting sequences (see Supplementary Information, Sup. Table 6). Of course, these methods need to be carefully analyzed and validated with other examples and comparisons. However, a clear indication seems to emerge about the applicability of informational indexes and laws, possibly after suitable improvements to support and complement the development of genome synthesis and analysis, in the spirit of new trends in synthetic biology.

The starting point of our investigation was the comparisons of real genomes with random genomes of the same length. To accomplish this purpose, the right length of *k*-mers equal to the double logarithmic length of genomes was identified as being more appropriate for this comparison because for this length random genomes reach their maximum entropy. The difference between entropies was considered a measure of the order acquired by real genomes and corresponded to their capability of realizing biological functions. This intuition was supported by the values of indexes that we computed for an initial list of genomes. In fact, Sup. Table 3 in Supplementary Information provides *AC* values that, apart from two evident exceptions, seem to confirm the increasing of the *AC* value in accordance with the macroscopic biological complexity of organisms (independently from length, number of genes, or other typical genomic parameters). However, when we extended our analysis by including other genomes^43^, we found *AC* values that were anomalous with respect to those already collected. In particular, plants provided extreme values, with no coherence with our interpretation of the *AC* index. To solve this puzzle, we considered a more comprehensive framework where *AC* and *EC* values interact in a trade-off between order and randomness. Genomes deviate from randomness, though to some extent, because genomes need a level of randomness that is sufficient to keep their evolutionary nature, based on a random exploration of new possibilities of life (filtered by natural selection).

In this picture, the two quantities 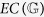 and 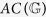 seem to correspond to the informational measure of two important aspects of genomes: *evolvability* and *programmability* (in the sense of^2^). Evolvability measures the random component of genomes, whereas programmability measures the order that genomes gain with respect to pure random genomes by acquiring biological functions. The non-random meaning of *AC* can be mathematically characterized in terms of Kullback-Leibler entropic divergence between the probability distribution of words of 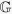 and the probability distribution of the same words in random genomes^44^.

Genome evolution is realized through an interplay of programmability and evolvability. The anti-entropic component *AC* cannot increase beyond a percentage of the logarithmic length because *LG* = *AC* + *EC* and therefore increase of *AC* implies a decrease of *EC* by reducing the evolutionary ability. Therefore, the only way to increase *AC*, by keeping a good balance of the two components, is to increase the value of *LG*, i. e., the genome length, which explains why genomes increase their length during evolution. However, this increase is only indirectly correlated with biological complexity, as apparent in Fig. 1 (see also Supplementary Information, Sup. Table 3).

The definition of genomic complexity, in terms of a nonlinear function of *AC*, is related to the balance between *AC* and *EC* values. Some of the genome entropic laws continue to also hold for *k*-mers with 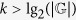, but almost none of the laws continue to hold when 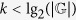. For example, for *k* = 6 and 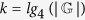, the values of *AC* completely lose the logic that they have for 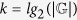, by showing dramatic changes with respect to 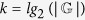, on which our indexes are based (see Supplementary Information, Sup. Table 4). Of course, we could compare real and random genomes also for values shorter than 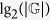, but in this case, we need to generate random genomes and compute the corresponding entropies, whereas for 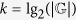, we do not need such generations and computations, because we know, by theoretical arguments (see Proposition 3) that in random genomes, entropies at double logarithmic lengths can be assumed to be equal to 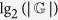.

Our investigation can be compared to the astronomical observations measuring positions and times in the orbits of celestial objects. Kepler’s laws arose from the regularities found in planetary motions, and from Kepler’s laws, the laws of mechanics emerged. This astronomical comparison, which was an inspiring analogy, revealed a surprising coincidence when ellipses were introduced in the representation of entropic and anti-entropic components. Kepler’s laws were explained by Newton’s dynamical and gravitational principles. Continuing our analogy, probably deeper informational principles are the ultimate reason for the laws that we found.

## Methods

The seventy investigated genomes include prokaryotes, algae, amoebae, fungi, plants, and animals of different types. In Sup. Table 5 of Supplementary Information, source data bases, assembly identifiers, genome lengths, and percentages of unknown nucleotides are given. Basic concepts from information theory, probability theory, and formal language theory can be found in classical texts in these fields^5^,^45^,^46^.

### Basic definitions and notation

Strings are finite sequences of contiguous symbols. Mathematically, strings are functions from a set of positions, viewed as a subset of the set 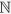 of natural numbers, 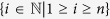 to a set of symbols, called *alphabet*. The number *n* is called the length of the string. We denote generic strings with Greek letters (possibly with subscripts) and reserve *λ* for the empty string (useful for expressing mathematical properties of strings). The length of a string *α* is denoted by |*α*|, and *α*[*i*] is the symbol occurring in *α* at position *i*, whereas *α*[*i*, *j*] is the string occurring in *α* between the positions *i* and *j* (both included).

Let us consider the genomic alphabet of four symbols (characters, or letters, associated with nucleotides) {*a*, *c*, *g*, *t*}. The set {*a*, *c*, *g*, *t*}*, as usual, denotes the set of all possible strings over {*a*, *c*, *g*, *t*}. A genome 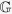 is representable by a string of {*a*, *c*, *g*, *t*}*, where symbols that occur, from the first to the last position, are written in the order that they occur, from left to right, according to the standard writing system of Western languages, and according to the chemical orientation 5′–3′ of DNA molecules.

Substrings 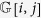 of length *k*, where 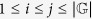, are also called *k-words*, *k-factors*, *k-mers* of 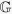 (*k* may be omitted, when it is not relevant). We remark that the absolute value notation |−| used for string length has different meaning when applied to sets or multisets. In fact, for a finite set *A*, then |*A*| denotes its cardinality, whereas for a finite multiset *X* (set of elements that possibly occur in many “identical” copies, with no relevance for occurrence order) |*X*| denotes its size (the sum of the elements of *X* each counted all the times that the element occurs).

A *dictionary of* 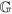 is a set of strings occurring in 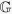. We denote by 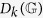 the dictionary of all *k*-mers occurring in *G*. It is easy to verify that the number of occurrences of *k*-mers in 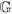 is 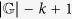 (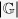 is the length of 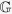) and corresponds to the maximum cardinality 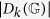 reachable by a dictionary of *k*-mers within genomes of the same length of 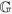.

A word *α* of *D* can occur in 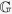 many times. We denote by 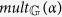 its *multiplicity* in 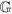, that is, the number of times *α* occurs in 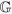. A word of 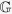 with multiplicity greater than 1 is called a *repeat* of 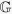, whereas a word with multiplicity equal to 1 is called a *hapax* of 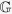. This term is used in philological investigation of texts, but it is also adopted in document indexing and compression^37^. The values of word multiplicities can be normalized if we divide the word multiplicities by the sum of the multiplicities of all the words occurring in 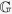. This normalization corresponds to replacing multiplicities with frequencies, which can be seen as percentages of multiplicity.

Many important indexes related to characteristics of genome dictionaries can be defined on genomes. For example, 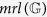 is the length of the longest repeats of 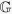. Of course, 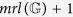 is the minimum length, such that *k*-mers with *k* greater than 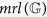 are all hapaxes.

Shannon used the term *information source* as synonymous with discrete probability distribution to introduce the notion of (information) *entropy*. Given a distribution of probability *p*, over a finite set *A*, its entropy is given by 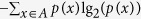. We remark that if −lg_2_(*p*(*x*)) is considered to be the information associated with the occurrence of *x*∈*A* (the more improbable *x* is, the more its occurrence is informative), then entropy is the mean (in a probabilistic sense) quantity of information emitted by the information source (*A*, *p*).

An intrinsic property of entropy is its *Equipartition Property*, that is, in the finite case, the fact that entropy reaches its maximum value lg_2_(|*A*|), when *p* is equally distributed, that is, when *p*(*x*) = 1/|*A*|, for all *x* ∈ *A* (|*A*| is the number of elements of *A*).

A genome 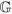 is any sequence over the alphabet {*a*, *c*, *g*, *t*}. This definition includes real genomes and ideal genomes, with no biological meaning, which are important in the mathematical analysis of genomes, as “material points” are essential in physics for discovering motion laws. Any subsequence of contiguous symbols of 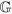 is called a string, word, or *k*-mer of 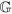 (*k* explicitly expresses the length).

The *empirical k-entropy* 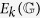 of 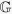 is given by (the adjective empirical refers to the use of frequencies):


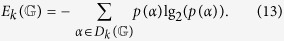


We remark that the entropy 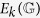 is computed only with the *k*-mers occurring in 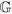 (see definition of 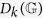). The computation of 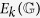 becomes prohibitive when 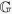 has length of order 10^9^ and *k* > 20. Therefore, we used suffix arrays^47^ in the computation of genomic dictionaries.

A *Bernoullian*, or random, genome is a synthetic genome generated by means of casual (blind) extractions (with insertion after extraction) from an urn containing four types of balls, in equal numbers of copies, completely identical apart from their colors, denoted by the genomic letters *a*, *c*, *g*, *t*. Pseudo-Bernoullian genomes can be generated by means of (pseudo) random generators available in programming languages (by suitable encoding of genomic symbols). We denote by *RND*_*n*_ the class of Bernoullian genomes of length *n*.

The computations of the main informational indexes, given in Table 1, extract the set of 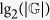-mers occurring in the considered genomes, where 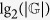 varies from 16 to 36, by means of a dedicated software, based on suffix arrays, called InfoGenomics Tools (shortly IGTools)^31^, which is an efficient suite of interactive tools mainly designed for extracting *k*-dictionaries, computing on them distributions and set-theoretic operations, and finally evaluating empirical entropies *E*_*k*_, and informational indexes, for different and even very large values of *k*.

In Supplementary Information, a 3D-visualization (Sup. Fig. 2) of 70 genomes is given with respect to *BB*, *AC*, *LX* axes, where Principal Component Analysis is applied for a better visualization. A taxonomy tree of the 70 genomes has been built via the NCBI taxonomy^48^ (see Supplementary Information, Sup. Fig. 1).

### Mathematical Backgrounds

In the following, some propositions are given, which were essential to the identification of parameters on which information entropies are computed. Let us start with the following question. Given a genome length *n* and a value *k* ≤ *n*, which is the maximum value of 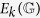 in the class of genomes of length *n*? We answer to the question above with Proposition 3, which is based on two Lemmas.

**Lemma 1** *Given a genome* 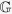 *of length n*, *if* 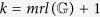, *then* 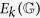 *is the maximum value that E*_*k*_ *can reach in the class of all possible genomes of length n*.

**Proof.** The minimum value of *k* such that all *k*-mers are hapaxes of 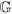 is 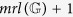. Therefore, if 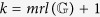, then 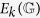 is maximum, according to the entropy Equipartition Property, because we have the maximum number of words occurring once in 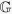, and all these words have the same probability of occurring in 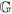. ☐

**Lemma 2** *If R is a random genome of length n*, *then*


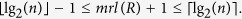


**Proof.** Let *RND*_*n*_ the class of random genomes of length *n*. If *k* = *mrl*(*R*) + 1, the probability that a *k*-mer occurs in *R*∈*RND*_*n*_ is (*n* − *k* + 1)/4^*k*^, and the probability that it occurs exactly once in *R* (being all *k*-mer hapaxes) is 1/(*n* − *k* + 1). Therefore, by equating these two probabilities we get:


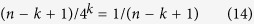


that is:


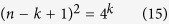


that implies (*k* has to be an integer) that the minimum length *k* for having all hapaxes in *R* is:


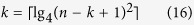


whence


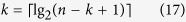


that is


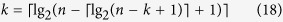


therefore


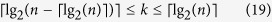


that implies the asserted inequality.☐

Table 2 shows an experimental validation of Lemma 2. It confirms that lg_2_(|*R*|) results to be a good estimation of the average of *mrl*(*R*) + 1 in 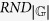.

**Proposition 3** *In the class of genomes of length n*, *for every k* < *n*, *the following relation holds*


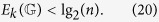


*Moreover, random genomes of length n have entropies differing from the upper bound* lg_2_(*n*) *less than* 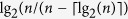 (*close to zero*).

**Proof.** According to Lemma 1, 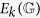 reaches its maximum, when 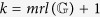. In this case:


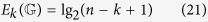


therefore, the difference 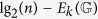 is given by:


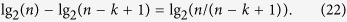


If 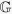 belongs to the class of random genomes of length *n*, according to Lemmas 1 and 2, the maximum entropy is given by 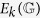, for 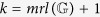, with 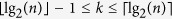. Therefore, by substituting in equation (22) the upper bound of *k*, giving the upper bound of lg_2_(*n*/(*n* − *k* + 1)), we get: 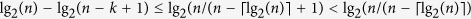.☐

## Additional Information

**How to cite this article**: Bonnici, V. and Manca, V. Informational laws of genome structures. *Sci. Rep*. **6**, 28840; doi: 10.1038/srep28840 (2016).

## Supplementary Material

Supplementary Information

## Figures and Tables

**Table 1:** Main informational genomic indexes.

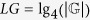	=	Logarithmic length
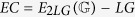	=	Entropic component
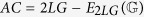	=	anti-entropic component
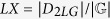	=	Lexical index
*AF* = *AC*/*LG*	=	anti-entropic fraction
*EH* = (*EC* − *AC*)/*LG*	=	Horizontal eccentricity
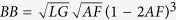	=	Biobit

|*D*_2*LG*_| is the number of 2*LG*-mers occurring in 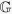, and 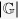 is the length of 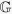.

**Table 2:** For each genome length, 100 trials were performed.

*length*	*min*	*max*	*sd*	*avg*	*lg*_2_(|*R*|)
1,000	9	15	1.07	10.2	9.97
100,000	15	20	0.95	16.67	16.61
200,000	16	21	0.86	17.78	17.61
500,000	18	23	0.91	19.09	18.93
1,000,000	18	24	0.96	20.14	19.93
10,000,000	22	26	0.97	23.49	23.25
20,000,000	23	27	0.93	24.31	24.25
30,000,000	24	30	1.14	25.08	24.84
50,000,000	24	31	1.17	25.86	25.58
75,000,000	25	29	0.85	26.44	26.16
100,000,000	25	30	1.02	26.89	26.58

The minimum, the maximum and the average, together with the standard deviation, of *mrl* + 1 was computed for each trial set. With a good approximation lg_2_(|*R*|) ≈ *avg*(*mrl*(*R*) + 1).
