# Author Correction: Informational laws of genome structures

**DOI:** 10.1038/s41598-023-30108-x

**Published:** 2023-02-28

**Authors:** Vincenzo Bonnici, Vincenzo Manca

**Affiliations:** 1 Department of Computer Science, University of Verona, Verona, 37134 Italy; 2 Center for BioMedical Computing, University of Verona, Verona, 37134 Italy

Correction to: *Scientific Reports* 10.1038/srep28840, published online 29 June 2016

This Article contains errors in the Methods section, subheading ‘Mathematical Backgrounds’.

Formulation of Lemma 1 is incorrect:

“Given a genome ${\mathbb {G}}$ of length *n*, if $k = mrl ({\mathbb {G}})+ 1$, then $E_k({\mathbb {G}})$ is the maximum value that $E_k$ can reach in the class of all possible genomes of length *n*”.

should read:

“Given a genome ${\mathbb {G}}$ of length n, if $k = mrl ({\mathbb {G}})+ 1$, then $E_k(G)$ is the maximum in the class of values $\{E_h(G) | n \ge h \ge k\}$”.

The corresponding proof:

“The minimum value of *k* such that all *k*-mers are hapaxes of ${\mathbb {G}}$ is $mrl({\mathbb {G}}) + 1$. Therefore, if $k = mrl ({\mathbb {G}})+ 1$, then $E_k({\mathbb {G}})$ is maximum, according to the entropy Equipartition Property, because we have the maximum number of words occurring once in ${\mathbb {G}}$, and all these words have the same probability of occurring in ${\mathbb {G}}$”.

should read:

“For $k = mrl +1$ empirical entropy $E_k(G)$ is equal to $\log _2(n-k+1)$, in fact $(n-k+1)$ is the number of distinct *k*-mers in ${\mathbb {G}}$. The same expression holds for any $h \le n$ and $h \ge k$, because string longer than *k* are hapaxes too. But, if $h > k$, then $\log _2(n-k+1) > \log _2(n-h+1)$, therefore $E_k(G) = max \{E_h(G) | n \ge h \ge k\}$”.

The proof of Lemma 2 is incorrect for the lack of an explicit characterization of “random genomes”. Here a correct proof is given:

First at all, a random genome of length *n* is obtained by a random process of generation where, at each step, one of four possible genome symbols is generated with probability 1/4. Let $k = mrl+1$. According to the theory of de Brujin sequences, it is possible to arrange all $4^k$ possible *k*-mers in a circular sequence $\alpha$ (the last symbol of $\alpha$ is followed by the first symbol of $\alpha$) where each *k*-mer occurs exactly once. Of course, any contiguous portion long n of $\alpha$ contains $(n-k+1)$ consecutive *k*-mers and corresponds to a random genome of length n (shortly, a *n*-genome). In fact, all symbols of $\alpha$ are equiprobable, and this homogeneity holds along all positions of $\alpha$, in the sense that, going forward (circularly) a number of steps equal to the length of $\alpha$ another de Brujin sequence, with the same equiprobability property is obtained, Let us consider the disjoint n-genomes (with no common k-mer) concatenated in $\alpha$. Their number is $m = 4^k/(n-k+1)$. But $\alpha$ is the shortest circular string arranging all *k*-mers, then, maximum statistical homogeneity (required by randomness) is reached when $m = (n-k+1)$, that is, when the probability that a *k*-mer has of occurring in one of the disjoint *n*-genomes of $\alpha$ is the same of occurring in one of the $(n-k+1)$ *k*-mer positions of a *n*-genome (a sort of scale-free equiprobability). This condition is expressed by equation (14) of the paper, from which equation $4^k = (n-k+1)^2$ follows, corresponding to equation (15). Whence, equations (16), (17), (18) and the inequality (19) follow, from which the bounds given for $mrl+1$ derive.

Consequently, proposition 3’s opening sentence:

“In the class of genomes of length n, for every $k < n$, the following relation holds:”

should read:

“In the class of genomes of length n, for every $mrl+1 \le k \le n$, the following relation holds:”

Finally, in Results, subheading ‘Information genomics laws’ Eq. 7 should be removed, because it follows from (8), being $LX >1$.

