# Supplementary material for: Informational laws of genome structures
Source: Sci Rep. 2016 Jun 29;6:28840. doi: 10.1038/srep28840 (PMC4937431; doi:10.1038/srep28840)
Supplement: Supplementary Information [file srep28840-s1.pdf]

Supplementary Information for

Informational laws of genome structures

Vincenzo Bonnici[1,2] **and Vincenzo Manca**[1,2,*]

[1]University of Verona, Department of Computer Science, University of Verona, Verona 37134, Italy
[2]Center for BioMedical Computing, University of Verona, Verona, 37134, Italy
[*]vincenzo.manca@univr.it

ABSTRACT

Figures and tables, supplementary to the main papers, are given that concern the 70 investigated genomes. Table 5 presents the essential data and the public databases where genomes were downloaded. Table 6 reports experiments in computational synthetic biology.

Table of Content

Figure 1: Taxonomy tree of the 70 genomes (Sup. Fig. 1) 2

Figure 2: A 3D-visualization of 70 genomes (axes AC, LX, BB) by means of Principal Component Analysis (Sup. Fig. 2) 3

Figure 3: The partition of LG represented by means of ellipses (Sup. Fig. 3) 3

Table 1: Horizontal Eccentricity Classes (Sup. Table 1) 4

Table 2: AC values for a kernel of 40 genomes (Sup. Table 2) 5

Table 3: Informational indexes for the 70 genomes (Sup. Table 3) 6

Table 4 Entropies and indexes for different values of k (Sup. Table 4) 7

Table 5 Basic data of the 70 genomes (Sup. Table 5) 8

Table 6 Computational experiments on synthetic genomes (Sup. Table 6) 9

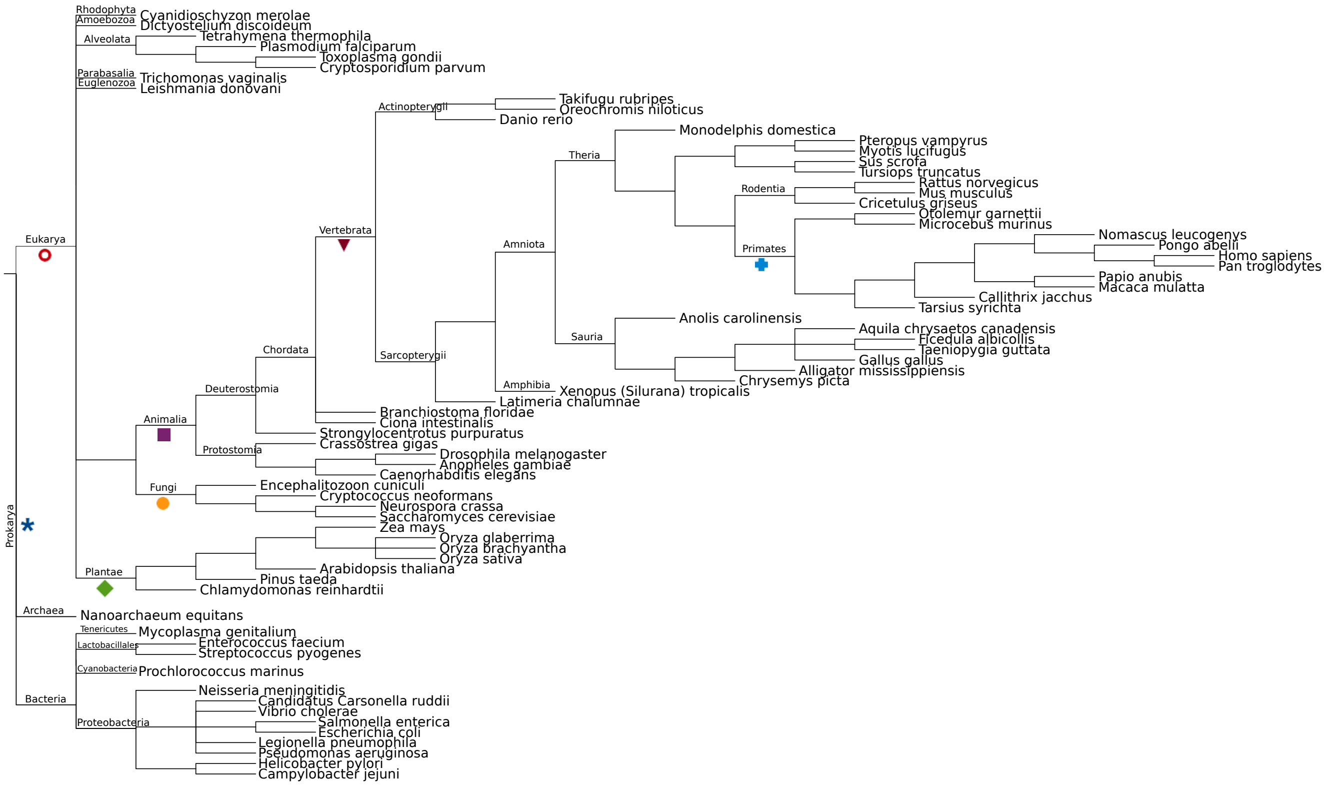

Figure 1. A taxonomy tree of the 70 analysed genomes. The has been built via the NCBI taxonomy (see reference 40 on the main paper) on-line service (*http://www.ncbi.nlm.nih.gov/Taxonomy/CommonTree/wwwcmt.cgi*). Branches of interest (Prokarya, Eukarya, Plantae, Fungi, Animalia, Vertebrata and Primates) are marked with their corresponding icon. Branches of the tree have fixed length, as opposed to classical phylogenetic trees where they length is based upon genetic distances.

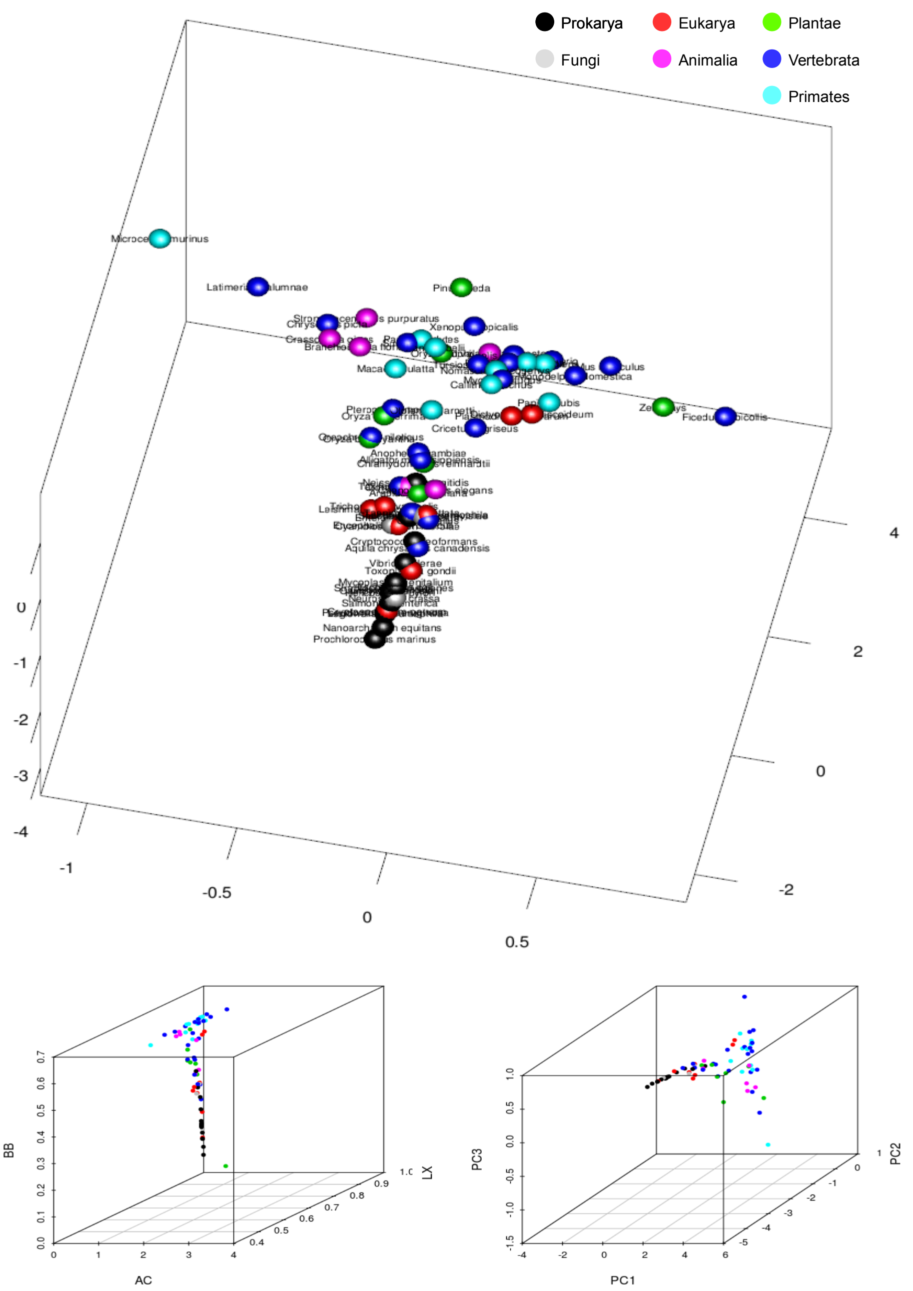

Figure 2. A 3D-visualization of 70 investigated genomes, obtained by taking into account three informational indexes AC, LX, BB. Bottom-left: the raw values of the three variables are plotted in 3D; Bottom-right: along the three axes Principal Component Analysis (PCA) of values on the left are used; Top: an enlarged version of Bottom-right is given, where a sort of tree (with exceptions) is apparent, going bottom-up (from primitive to more complex genomes).

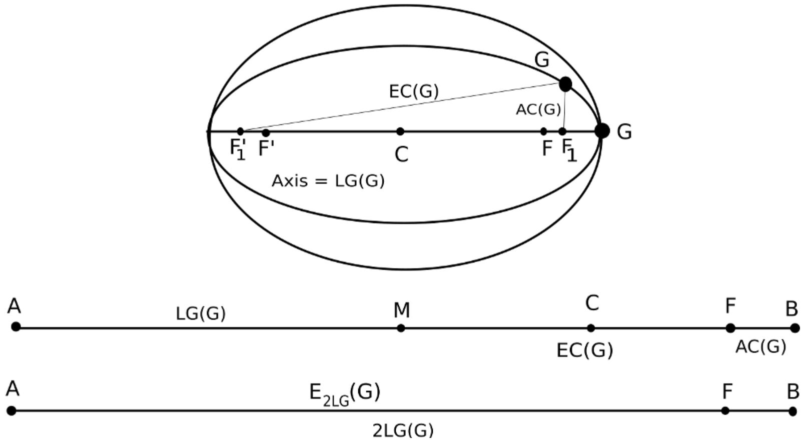

Figure 3. The partition of genome logarithmic length and the horizontal and vertical genomic ellipses. Segment AB corresponds to $2LG$, segments AM, MB correspond to LG, segment AF corresponds to E_{2LG}, segment MF and FB correspond to EC and AC, respectively, and F determines a partition of LG. Point F correspond to a focus of the horizontal ellipse, while F_1 to a focus of the vertical ellipse, and C is the center of ellipses. The less eccentric ellipse is the *horizontal genomic ellipse*, where $\mathbb{G}$ is on the major axis, while the more eccentric one is the *vertical genomic ellipse*, where $\mathbb{G}$ is on the line orthogonal to the major axis and passing on its focus. The eccentricity EV of the vertical ellipse is the root square of the horizontal eccentricity EH, and the distance between the focuses F and F_1 is $EV(1-EV)LG/2$. Infinite ellipses, with the same major axis, but with different values of eccentricity, can represent $\mathbb{G}$ as a point placed at different angles from the axis. The genomic complexity measure $BB(\mathbb{G})$, *biobit* of $\mathbb{G}$, is defined by means of the horizontal eccentricity EH.

First Class	Eccentriciy	Range 0.9990-0.9400
Prochlorococcus marinus	0.9991	
Nanoarchaeum equitans	0.9978	
Pseudomonas aeruginosa	0.9969	
Legionella pneumophila	0.9969	
Cryptosporidium parvum	0.9969	
Salmonella enterica	0.9956	
Neurospora crassa	0.9956	
Helicobacter pylori	0.9956	
Campylobacter jejuni	0.9934	
Escherichia coli	0.9931	
Streptococcus pyogenes	0.9928	
Carsonella ruddii	0.9919	
Mycoplasma genitalium	0.9910	
Toxoplasma gondii	0.9909	
Aquila chrysaetos canadensis	0.9883	
Vibrio cholerae	0.9879	
Cryptococcus neoformans	0.9838	
Gallus gallus	0.9819	
Taeniopygia guttata	0.9812	
Cyanidioschyzon merolae	0.9807	
Leishmania donovani	0.9794	
Encephalitozoon cuniculi	0.9782	
Tetrahymena thermophila	0.9781	
Saccharomyces cerevisiae	0.9754	
Enterococcus faecium	0.9748	
Takifugu rubripes	0.9733	
Arabidopsis thaliana	0.9725	
Ciona intestinalis	0.9708	
Caenorhabditis elegans	0.9694	
Trichomonas vaginalis	0.9691	
Alligator mississippiensis	0.9665	
Oreochromis niloticus	0.9605	
Oryza brachyantha	0.9591	
Anopheles gambiae	0.9588	
Neisseria meningitidis	0.9585	
Pteropus vampyrus	0.9498	
Cricetulus griseus	0.9470	
Oryza glaberrima	0.9464	
**Second Class**	Eccentriciy	Range 0.9300-0.8400
Chlamydomonas reinhardtii	0.9303	
Macaca mulatta	0.9264	
Papio anubis	0.9190	
Callithrix jacchus	0.9178	
Myotis lucifugus	0.9086	
Crassostrea gigas	0.9049	
Chrysemys picta	0.9042	
Branchiostoma floridae	0.9041	
Nomascus leucogenys	0.9040	
Tursiops truncatu	0.9023	
Plasmodium falciparum	0.9015	
Sus scrofa	0.9006	
Pongo albelii	0.8989	
Dictyostelium discoideum	0.8964	
Pan troglodytes	0.8948	
Rattus norvegicus	0.8919	
Branchiostoma floridae	0.8895	
Tarsius syrichta	0.8878	
Monodelphis domestica	0.8875	
Latimeria chalumnae	0.8857	
Homo sapiens	0.8817	
Oryza sativa	0.8790	
Strongylocentrotus purpuratus	0.8711	
Ficedula albicollis	0.8684	
Anolis carolinensis	0.8647	
Danio rerio	0.8581	
Mus musculus	0.8504	
Microcebus murinus	0.8499	
**Third Class**	Eccentriciy	Range 0.8300-0.7300
Xenopus tropicalis	0.8260	
Drosophila melanogaster	0.8173	
**Fourth Class**	Eccentriciy	Range 0.7400-0.5200
Pinus taeda	0.7450	
Zea mays	0.5258	

Table 1. Classes of horizontal genomic eccentricity. The classes are clearly divided by jumps between extremal values of the four eccentricity classes.

Species	$LG(\mathbb{G})$	$N\%$	Genes	$AC(\mathbb{G})$
Carsonella ruddii	8.642	0.00	0.1k	0.035
Chlamydomonas reinhardtii	8.872	0.00	14.4k	0.309
Mycoplasma genitalium	9.573	0.00	0.5k	0.043
Trichomonas vaginalis	9.579	0.36	60.8k	0.148
Campylobacter jejuni	10.323	0.00	1.6k	0.034
Helicobacter pylori	10.335	0.00	1.5k	0.033
Prochlorococcus marinus	10.370	0.00	1.9k	0.005
Streptococcus pyogenes	10.410	0.00	1.8k	0.037
Neisseria meningitidis	10.558	0.00	2.1k	0.219
Encephalitozoon cuniculi	10.626	0.03	2.0k	0.115
Enterococcus faecium	10.771	0.00	3.2k	0.135
Caenorhabditis elegans	13.290	0.00	46.6k	0.203
Tetrahymena thermophila	13.309	0.06	26.9k	0.145
Arabidopsis thaliana	13.417	0.16	33.5k	0.184
Oryza brachyantha	13.951	6.43	24.2k	0.286
Takifugu rubripes	14.035	4.52	17.3k	0.187
Oryza glaberrima	14.043	4.00	73.8k	0.376
Oryza sativa	14.256	2.63	30.5k	0.862
Branchiostoma floridae	14.480	7.95	28.6k	0.694
Crassostrea gigas	14.527	11.81	32.2k	0.690
Oreochromis niloticus	14.646	7.74	22.6k	0.289
Taeniopygia guttata	14.964	0.64	14.1k	0.140
Pteropus vampyrus	15.447	7.85	33.3k	0.397
Myotis lucifugus	15.461	3.35	44.1k	0.706
***Zea mays	15.470	0.63	52.2k	3.668
Cricetulus griseus	15.568	2.49	26.1k	0.412
Tursiops truncatus	15.624	8.60	23.7k	0.763
Chrysemys picta	15.635	16.66	23.6k	0.748
Sus scrofa	15.637	10.52	35.5k	0.776
Papio anubis	15.672	1.54	38.6k	0.635
Mus musculus	15.672	2.86	46.2k	1.172
Callithrix jacchus	15.684	5.38	33.2k	0.644
Rattus norvegicus	15.687	4.68	37.2k	0.847
Nomascus leucogenys	15.690	6.57	27.3k	0.753
Latimeria chalumnae	15.707	23.67	26.1k	0.897
Pongo abelii	15.748	10.12	29.7k	0.796
Homo sapiens	15.762	4.88	50.7k	0.932
Pan troglodytes	15.776	12.41	33.1k	0.829
Tarsius syrichta	15.843	1.39	25.9k	0.888
Monodelphis domestica	15.853	2.56	34.8k	0.891
***Pinus taeda	17.216	13.08	50.1k	2.194

Table 2. *AC* values for forty different species. Length of genomes, *N%* (the percentage of unknown nucleotides), and number of genes (in the wider sense of transcribed regions, that is, protein and non-protein coding genes) are not related to *AC* values. Color red and *** mark two cases of anomalous *AC* values. Finding a genomic complexity measure based on *AC*, but overcoming the anomalies of two marked cases, was the starting point of the logarithmic length partition idea and of informational indexes based on it.

Species	$LG(\mathbb{G})$	$EC(\mathbb{G})$	$AC(\mathbb{G})$	$LX(\mathbb{G})$	$AF(\mathbb{G})$	$EH(\mathbb{G})$	$BB(\mathbb{G})$
Carsonella ruddii	8.6423	8.6069	0.0355	1.0158	0.0041	0.9918	0.1837
Chlamydomonas reinhardtii	8.8722	8.5628	0.3094	1.0967	0.0349	0.9302	0.4478
Nanoarchaeum equitans	9.4525	9.4429	0.0097	1.0029	0.0010	0.9980	0.0977
Mycoplasma genitalium	9.5729	9.5302	0.0428	1.0177	0.0045	0.9911	0.2013
Trichomonas vaginalis	9.5790	9.4314	0.1476	1.0687	0.0154	0.9692	0.3497
Campylobacter jejuni	10.3233	10.2892	0.0341	1.0155	0.0033	0.9934	0.1809
Helicobacter pylori	10.3348	10.3022	0.0326	1.0147	0.0032	0.9937	0.1771
Prochlorococcus marinus	10.3699	10.3654	0.0045	1.0017	0.0004	0.9991	0.0671
Streptococcus pyogenes	10.4105	10.3731	0.0374	1.0157	0.0036	0.9928	0.1893
Neisseria meningitidis	10.5579	10.3391	0.2187	1.0752	0.0207	0.9586	0.4119
Encephalitozoon cuniculi	10.6260	10.5106	0.1155	1.0518	0.0109	0.9783	0.3181
Enterococcus faecium	10.7708	10.6355	0.1353	1.0504	0.0126	0.9749	0.3408
Legionella pneumophila	10.8481	10.8310	0.0171	1.0075	0.0016	0.9969	0.1295
Vibrio cholerae	10.9718	10.9058	0.0660	1.0238	0.0060	0.9880	0.2477
Salmonella enterica	11.1197	11.0954	0.0243	1.0099	0.0022	0.9956	0.1540
Escherichia coli	11.1318	11.0936	0.0382	1.0153	0.0034	0.9931	0.1914
Pseudomonas aeruginosa	11.2894	11.2719	0.0175	1.0079	0.0015	0.9969	0.1310
Cryptosporidium parvum	11.5589	11.5403	0.0186	1.0079	0.0016	0.9968	0.1350
Saccharomyces cerevisiae	11.7676	11.6235	0.1442	1.0455	0.0123	0.9755	0.3525
Cyanidioschyzon merolae	11.9156	11.8012	0.1144	1.0488	0.0096	0.9808	0.3191
Cryptococcus neoformans	12.0917	11.9943	0.0974	1.0323	0.0081	0.9839	0.2972
Plasmodium falciparum	12.2360	11.6337	0.6023	1.1290	0.0492	0.9016	0.5687
Leishmania donovani	12.4757	12.3473	0.1285	1.0707	0.0103	0.9794	0.3367
Dictyostelium discoideum	12.5086	11.8609	0.6477	1.1280	0.0518	0.8964	0.5798
Neurospora crassa	12.6362	12.6070	0.0292	1.0104	0.0023	0.9954	0.1685
Toxoplasma gondii	12.9402	12.8819	0.0583	1.0170	0.0045	0.9910	0.2350
Ciona intestinalis	13.1112	12.9199	0.1913	1.0735	0.0146	0.9708	0.4002
Caenorhabditis elegans	13.2898	13.0865	0.2033	1.0598	0.0153	0.9694	0.4108
Tetrahymena thermophila	13.3091	13.1641	0.1451	1.0443	0.0109	0.9782	0.3565
Arabidopsis thaliana	13.4172	13.2330	0.1842	1.0635	0.0137	0.9725	0.3948
Drosophila melanogaster	13.6651	12.4174	1.2477	1.3403	0.0913	0.8174	0.6100
Anopheles gambiae	13.8900	13.6037	0.2863	1.0990	0.0206	0.9588	0.4716
Oryza brachyantha	13.9513	13.6657	0.2857	1.1309	0.0205	0.9590	0.4715
Takifugu rubripes	14.0345	13.8478	0.1867	1.0757	0.0133	0.9734	0.3985
Oryza glaberrima	14.0433	13.6672	0.3761	1.1523	0.0268	0.9464	0.5199
Oryza sativa	14.2560	13.3940	0.8619	1.2650	0.0605	0.8791	0.6307
Branchiostoma floridae	14.4796	13.7859	0.6937	1.2802	0.0479	0.9042	0.6157
Crassostrea gigas	14.5275	13.8372	0.6903	1.3060	0.0475	0.9050	0.6158
Oreochromis niloticus	14.6461	14.3569	0.2891	1.1319	0.0197	0.9605	0.4765
Strongylocentrotus purpuratus	14.9421	13.9797	0.9624	1.3713	0.0644	0.8712	0.6486
Gallus gallus	14.9515	14.8165	0.1349	1.0401	0.0090	0.9820	0.3478
Taeniopygia guttata	14.9640	14.8239	0.1401	1.0515	0.0094	0.9813	0.3537
Ficedula albicollis	14.9798	13.9946	0.9852	1.1524	0.0658	0.8685	0.6502
Anolis carolinensis	15.0053	13.9903	1.0150	1.2547	0.0676	0.8647	0.6514
Aquila chrysaetos canadensis	15.0758	14.9877	0.0881	1.0270	0.0058	0.9883	0.2866
Danio rerio	15.1600	14.0845	1.0755	1.2414	0.0709	0.8581	0.6553
Xenopus tropicalis	15.2468	13.9207	1.3261	1.3937	0.0870	0.8260	0.6491
Pteropus vampyrus	15.4473	15.0499	0.3973	1.1548	0.0257	0.9486	0.5380
Myotis lucifugus	15.4610	14.7547	0.7064	1.1734	0.0457	0.9086	0.6305
Zea mays	15.4701	11.8023	3.6678	2.3367	0.2371	0.5258	0.2784
Alligator mississippiensis	15.5089	15.2492	0.2597	1.0896	0.0167	0.9665	0.4601
Cricetulus griseus	15.5681	15.1565	0.4116	1.1037	0.0264	0.9471	0.5451
Otolemur garnetti	15.6153	15.1820	0.4333	1.1397	0.0277	0.9445	0.5546
Tursiops truncatus	15.6245	14.8615	0.7630	1.2088	0.0488	0.9023	0.6417
Chrysemys picta	15.6351	14.8867	0.7484	1.3332	0.0479	0.9043	0.6397
Sus scrofa	15.6370	14.8606	0.7764	1.2709	0.0497	0.9007	0.6438
Papio anubis	15.6716	15.0369	0.6347	1.1202	0.0405	0.9190	0.6183
Mus musculus	15.6719	14.4999	1.1720	1.2244	0.0748	0.8504	0.6659
Callithrix jacchus	15.6837	15.0393	0.6444	1.1633	0.0411	0.9178	0.6207
Rattus norvegicus	15.6867	14.8396	0.8471	1.2106	0.0540	0.8920	0.6532
Nomascus leucogenys	15.6902	14.9371	0.7531	1.1923	0.0480	0.9040	0.6411
Latimeria chalumnae	15.7068	14.8094	0.8974	1.4661	0.0571	0.8857	0.6583
Macaca mulatta	15.7134	15.1346	0.5788	1.2144	0.0368	0.9263	0.6047
Microcebus murinus	15.7173	14.5382	1.1790	1.7376	0.0750	0.8500	0.6668
Pongo abelii	15.7482	14.9524	0.7958	1.2542	0.0505	0.8989	0.6480
Homo sapiens	15.7621	14.8298	0.9322	1.2083	0.0591	0.8817	0.6618
Pan troglodytes	15.7756	14.9464	0.8292	1.2775	0.0526	0.8949	0.6526
Tarsius syrichta	15.8428	14.9545	0.8883	1.2094	0.0561	0.8879	0.6597
Monodelphis domestica	15.8528	14.9615	0.8913	1.1738	0.0562	0.8876	0.6601
Pinus taeda	17.2161	15.0218	2.1943	1.8132	0.1275	0.7451	0.6127

Table 3. The table of seventy genomes ordered by logarithmic length, LG, with the values of the main informational indexes.

Species	Entropy			Antientropic component		
	$k = lg_2(\lvert\mathbb{G}\rvert)$	$k = lg_4(\lvert\mathbb{G}\rvert)$	$k = 6$	$k = lg_2(\lvert\mathbb{G}\rvert)$	$k = lg_4(\lvert\mathbb{G}\rvert)$	$k = 6$
Carsonella ruddii	17.2492	13.4697	9.6804	0.0355	3.8149	7.6042
Chlamydomonas reinhardtii	17.4350	15.7458	11.3652	0.3094	1.9986	6.3793
Nanoarchaeum equitans	18.8954	16.7812	11.1303	0.0097	2.1238	7.7747
Mycoplasma genitalium	19.1031	17.0126	11.1289	0.0428	2.1333	8.0170
Trichomonas vaginalis	19.0103	17.2127	11.2786	0.1476	1.9452	7.8793
Campylobacter jejuni	20.6125	18.2061	11.0350	0.0341	2.4405	9.6116
Helicobacter pylori	20.6370	18.6428	11.3566	0.0326	2.0268	9.3129
Prochlorococcus marinus	20.7353	19.1064	11.5169	0.0045	1.6334	9.2229
Streptococcus pyogenes	20.7836	19.3124	11.6361	0.0374	1.5086	9.1848
Neisseria meningitidis	20.8970	19.2370	11.6142	0.2187	1.8788	9.5015
Encephalitozoon cuniculi	21.1366	19.8268	11.7543	0.1155	1.4252	9.4978
Enterococcus faecium	21.4063	19.8874	11.5896	0.1353	1.6542	9.9520
Legionella pneumophila	21.6791	20.2001	11.6195	0.0171	1.4960	10.0766
Vibrio cholerae	21.8776	20.0712	11.8176	0.0660	2.5076	10.1260
Salmonella enterica	22.2151	20.6806	11.7528	0.0243	1.2630	10.4866
Escherichia coli	22.2254	20.8378	11.7829	0.0382	1.4016	10.4807
Pseudomonas aeruginosa	22.5613	20.9072	11.2003	0.0175	1.3564	11.3784
Cryptosporidium parvum	23.0992	20.8029	11.1674	0.0186	2.3149	11.9504
Saccharomyces cerevisiae	23.3911	19.4624	11.6736	0.1442	5.0096	11.8617
Cyanidioschyzon merolae	23.7168	21.9660	11.8021	0.1144	1.5693	12.0292
Cryptococcus neoformans	24.0861	20.1987	11.8996	0.0974	4.8186	12.2838
Plasmodium falciparum	23.8697	22.4388	9.9978	0.6023	1.3924	14.4742
Leishmania donovani	24.8230	22.9899	11.6093	0.1285	1.1935	13.3422
Dictyostelium discoideum	24.3695	23.0664	10.2441	0.6477	1.8851	14.7731
Neurospora crassa	25.2432	23.8994	11.9033	0.0292	1.3730	13.3691
Toxoplasma gondii	25.8221	22.6411	11.7489	0.0583	3.9772	14.1315
Ciona intestinalis	26.0311	24.3322	11.5368	0.1913	1.5482	14.6856
Caenorhabditis elegans	26.3762	24.0225	11.4139	0.2033	2.1999	15.1656
Tetrahymena thermophila	26.4732	24.1318	10.4827	0.1451	2.4477	16.1356
Arabidopsis thaliana	26.6503	24.7988	11.5543	0.1842	2.0357	15.2801
Drosophila melanogaster	26.0825	24.6902	11.7565	1.2477	2.6400	15.5737
Anopheles gambiae	27.4937	25.9133	11.8110	0.2863	1.9894	15.9691
Oryza brachyantha	27.6170	26.0192	11.7356	0.2857	1.7609	16.1670
Takifugu rubripes	27.8824	26.0377	11.7420	0.1867	2.0488	16.3270
Oryza glaberrima	27.7104	26.3551	11.7984	0.3761	1.7139	16.2881
Oryza sativa	27.6500	25.9591	11.8117	0.8619	2.5529	16.7002
Branchiostoma floridae	28.2655	26.0147	11.7719	0.6937	2.7076	17.1873
Crassostrea gigas	28.3647	26.1425	11.4230	0.6903	2.9125	17.6319
Oreochromis niloticus	29.0030	27.1295	11.6116	0.2891	2.1626	17.6805
Strongylocentrotus purpuratus	28.9218	26.8875	11.6060	0.9624	2.9966	18.2782
Gallus gallus	29.7680	26.7651	11.5773	0.1349	3.2455	18.3256
Taeniopygia guttata	29.7879	27.0544	11.5050	0.1401	2.9052	18.4230
Ficedula albicollis	28.9744	27.7685	11.3629	0.9852	2.1595	18.5967
Anolis carolinensis	28.9956	26.5610	11.5850	1.0150	3.7590	18.4256
Aquila chrysaetos canadensis	30.0635	27.8182	11.6049	0.0881	2.0847	18.5467
Danio rerio	29.2445	25.1100	11.5093	1.0755	5.8302	18.8107
Xenopus tropicalis	29.1674	27.0775	11.6203	1.3261	3.4161	18.8733
Pteropus vampyrus	30.4972	28.1798	11.5986	0.3973	1.9718	19.2959
Myotis lucifugus	30.2157	27.9072	11.6182	0.7064	3.0149	19.3038
Zea mays	27.2725	28.4813	11.8822	3.6678	2.4133	19.0581
Alligator mississippiensis	30.7582	27.7725	11.6238	0.2597	3.6412	19.3941
Cricetulus griseus	30.7246	28.5329	11.5406	0.4116	2.4849	19.5956
Otolemur garnetti	30.7973	28.3040	11.5669	0.4333	2.8322	19.6637
Tursiops truncatus	30.4860	28.3923	11.6206	0.7630	3.0423	19.6284
Chrysemys picta	30.5218	27.9411	11.6751	0.7484	3.3329	19.5951
Sus scrofa	30.4976	28.5049	11.5885	0.7764	2.7653	19.6856
Papio anubis	30.7086	28.3383	11.5622	0.6347	2.8923	19.7811
Mus musculus	30.1719	28.2116	11.5680	1.1720	3.0373	19.7759
Callithrix jacchus	30.7230	27.8796	11.5562	0.6444	3.4643	19.8111
Rattus norvegicus	30.5264	28.0116	11.5934	0.8471	3.3688	19.7801
Nomascus leucogenys	30.6272	28.0905	11.5569	0.7531	3.2768	19.8234
Latimeria chalumnae	30.5162	28.0310	11.5712	0.8974	3.3123	19.8424
Macaca mulatta	30.8480	28.3075	11.5616	0.5788	3.0352	19.8652
Microcebus murinus	30.2555	28.3216	11.6011	1.1790	3.1051	19.8334
Pongo abelii	30.7006	28.1258	11.5518	0.7958	3.3706	19.9447
Homo sapiens	30.5919	28.2741	11.5572	0.9322	3.0994	19.9669
Pan troglodytes	30.7220	28.1484	11.5533	0.8292	3.4028	19.9978
Tarsius syrichta	30.7973	27.9580	11.5743	0.8883	3.5661	20.1113
Monodelphis domestica	30.8144	28.0452	11.4268	0.8913	3.6605	20.2789
Pinus taeda	32.2379	28.0957	11.5953	2.1943	3.5899	22.8368

Table 4. Entropy and antientropic component of the 70 analyzed genomes by taking into account $k = lg_2(|G|)$, $k = lg_4(|G|)$, and $k = 6$.

Species	Taxonomy	Common name	Source	ID	$\vert\mathbb{G}\vert$	$N\%$
Alligator mississippiensis	vertebrata	american alligator	UCSC	allMis1	2,174,243,242	2.09
Anolis carolinensis	vertebrata	carolina anole (lizard)	UCSC	anoCar2	1,081,661,814	5.14
Anopheles gambiae	animalia		NCBI	PEST	230,482,020	2.36
Aquila chrysaetos canadensis	vertebrata	golden eagle	NCBI	aquChr1	1,192,743,076	1.07
Arabidopsis thaliana	plantae		Ensembl	TAIR10	119,667,750	0.16
Branchiostoma floridae	animalia	lancet	NCBI	barFlo1	521,895,125	7.95
Caenorhabditis elegans	animalia		UCSC	ce10	100,286,070	0.00
Callithrix jacchus	primates	marmoset	UCSC	calJac3	2,770,219,215	5.38
Campylobacter jejuni	prokaryota		NCBI	NC_002163.1	1,641,481	0.00
Carsonella ruddii	prokaryota		NCBI	PV	159,662	0.00
Chlamydomonas reinhardtii	plantae	green alga	NCBI	v3.1.28	219,586	0.00
Chrysemys picta	vertebrata	painted turtle	UCSC	chrPic1	2,589,745,704	16.66
Ciona intestinalis	vertebrata		NCBI	KH	78,296,155	2.49
Crassostrea gigas	animalia	pacific oyster	Ensembl	GCA_000297895.1.25	557,717,710	11.81
Cricetulus griseus	vertebrata	chinese hamster	UCSC	criGri1	2,360,130,144	2.49
Cryptococcus neoformans	fungi		NCBI	JEC21	19,051,922	0.01
Cryptosporidium parvum	eukaryota		NCBI	II	9,102,324	0.20
Cyanidioschyzon merolae	eukaryota	red alga (single-cell)	Ensembl	ASM9120v1	14,925,130	0.00
Danio rerio	vertebrata	zebrafish	NCBI	GRCz10	1,340,430,591	0.14
Dictyostelium discoideum	eukaryota		NCBI	GCA_000004695.1	33,958,027	0.07
Drosophila melanogaster	animalia	fruit fly	UCSC	dm3	168,736,537	3.77
Encephalitozoon cuniculi	fungi		NCBI	GB-M1	2,497,519	0.03
Enterococcus faecium	prokaryota		NCBI	DO	3,052,572	0.00
Escherichia coli	prokaryota		NCBI	536	5,034,947	0.00
Ficedula albicollis	vertebrata	collared flycatcher	NCBI	AGTO02	1,044,082,078	0.88
Gallus gallus	vertebrata	red junglefowl (chicken)	UCSC	galGal4	1,003,853,215	1.08
Helicobacter pylori	prokaryota		NCBI	NC_000915.1	1,667,867	0.00
Homo sapiens	primates	human	UCSC	GRCh38	3,088,286,401	4.88
Latimeria chalumnae	vertebrata	west indian ocean coelacanth	UCSC	latCha1	2,860,591,921	23.67
Legionella pneumophila	prokaryota		NCBI	Philadelphia1	3,397,754	0.00
Leishmania donovani	eukaryota		NCBI	ASM22713v2	32,444,968	3.68
Macaca mulatta	primates	rhesus macaque	UCSC	rheMac3	2,886,669,826	11.21
Microcebus murinus	primates	grey mouse lemur	UCSC	micMur1	2,902,270,736	36.17
Monodelphis domestica	vertebrata	gray short-tailed opossum	UCSC	monDom5	3,502,390,117	2.56
Mus musculus	vertebrata	mouse	UCSC	mm10	2,725,537,669	2.86
Mycoplasma genitalium	prokaryota		NCBI	G37	580,076	0.00
Myotis lucifugus	vertebrata	little brown bat	UCSC	myoLuc2	2,034,575,300	3.35
Nanoarchaeum equitans	prokaryota		NCBI	Kin4-M	490,885	0.00
Neisseria meningitidis	prokaryota		NCBI	MC58	2,272,360	0.00
Neurospora crassa	fungi		NCBI	OR74A	40,527,912	0.09
Nomascus leucogenys	primates	northen white-cheeked gibbon	UCSC	nomLeu3	2,795,260,045	6.57
Oreochromis niloticus	vertebrata	nile tilapia	UCSC	oreNil2	657,367,599	7.74
Oryza brachyantha	plantae	african wild rice	Ensembl	oryza1.4b	250,927,218	6.43
Oryza glaberrima	plantae	african rice (domesticated)	Ensembl	AGI1.1	285,037,524	4.00
Oryza sativa	plantae	asian rice (common rice)	NCBI	v4.0	382,778,125	2.63
Otolemur garnetti	primates	northen greater galago (bush baby)	UCSC	otoGar3	2,519,724,550	6.36
Pan troglodytes	primates	chimpanzee	UCSC	panTro4	3,146,680,125	12.41
Papio anubis	primates	olive baboon	UCSC	papAnu2	2,724,327,674	1.54
Pinus taeda	plantae	loblolly pine	Dendrome	v1.01	23,180,477,227	13.08
Plasmodium falciparum	eukaryota		NCBI	3D7	23,270,305	0.00
Pongo abelii	primates	sumatran orangutan	UCSC	ponAbe2	3,029,507,528	10.12
Prochlorococcus marinus	prokaryota		NCBI	NC_005042.1	1,751,080	0.00
Pseudomonas aeruginosa	prokaryota		NCBI	PAO1	6,264,404	0.00
Pteropus vampyrus	vertebrata	large flying fox (big bat)	UCSC	pteVam1	1,996,076,410	7.85
Rattus norvegicus	vertebrata	brown rat	UCSC	rn6	2,782,028,915	4.68
Saccharomyces cerevisiae	fungi	brewer's yeast	UCSC	sacCer3	12,157,105	0.00
Salmonella enterica	prokaryota		NCBI	LT2	4,951,371	0.00
Streptococcus pyogenes	prokaryota		NCBI	M1GAS	1,852,433	0.00
Strongylocentrotus purpuratus	animalia	strongylocentrotus purpuratus (sea urchin)	NCBI	Spur4.2	990,915,289	8.93
Sus scrofa	vertebrata	wild boar (pig)	UCSC	susScr3	2,596,656,069	10.52
Taeniopygia guttata	vertebrata	zebra finch	UCSC	taeGut2	1,021,479,793	0.64
Takifugu rubripes	vertebrata		UCSC	fr3	281,588,809	4.52
Tarsius syrichta	primates	philippine tarsier	UCSC	tarSyr2	3,453,864,774	1.39
Tetrahymena thermophila	eukaryota		NCBI	SB210	103,014,375	0.06
Toxoplasma gondii	eukaryota		Ensembl	ME49	61,769,095	0.06
Trichomonas vaginalis	eukaryota		TrichDB	G3	584,929	0.36
Tursiops truncatus	vertebrata	common bottlenose dolphin	UCSC	turTru2	2,551,980,185	8.60
Vibrio cholerae	prokaryota		NCBI	N16961	4,033,464	0.00
Xenopus tropicalis	vertebrata	western clawed frog	UCSC	xenTro3	1,511,735,326	10.15
Zea mays	plantae	maize	NCBI	B73v3	2,060,411,742	0.63

Table 5. The complete list of 70 genomes with the common names (when available), their biological taxonomy, their common name, the source database from where each has been downloaded, the corresponding genome assembly ID within the database, the genome length $|G|$, (genomes may include standard chromosomes, as well as mitochondrial ones, plasmids and linkage groups),and the percentage $N\%$ of unknown nucleotides.

Gene ID	Description
MG_rrnA16S	16S ribosomal rRNA
MG_289	phosphonate ABC transporter, substrate binding protein (P37), putative
MG_340	DNA-directed RNA polymerase, beta subunit
MG_497	tRNA (ARG)
MG_186	Oligosaccharide or polyol ABC transporter, substrate binding protein
MG_194	phenylalanyl-tRNA synthetase, alpha subunit
MG_312	high molecular weight cytadherence accessory protein 1
MG_069	PTS system, glucose-specific IIABC component
MG_386	P200 protein
MG_191	MgPa adhesin
MG_217	P65 adhesin
MG_192	P110 protein
MG_318	P32 adhesin
MG_159	ribosomal protein L29
MG_317	high molecular weight cytadherence accessory protein 3
MG_338	lipoprotein, putative
MG_200	DnaJ domain protein
MG_203	DNA topoisomerase IV, B subunit
MG_226	amino acid-polyamine-organocation (APC) permease
MG_506	tRNA (SER)
MG_042	spermidine/putrescine (polyamine) ABC transporter, ATP-binding protein, putative
MG_185	lipoprotein, putative
MG_309	lipoprotein, putative
MG_509	tRNA (LYS)
MG_434	uridylate kinase
MG_079	oligopeptide ABC transporter, ATP-binding protein
MG_068	lipoprotein, putative
MG_470	CobQ/CobB/MinD/ParA nucleotide binding domain
MG_219	unknown
MG_290	phosphonate ABC transporter, ATP-binding protein, putative
MG_526	salivaricin A ABC transporter, ATP-binding protein
MG_384	GTPase1 Obg
MG_111	glucose-6-phosphate isomerase
MG_100	glutamyl-tRNA(Gln) and/or aspartyl-tRNA(Asn) amidotransferase, B subunit
MG_500	tRNA (LEU)
MG_246	Ser/Thr protein phosphatase 2A
MG_118	UDP-glucose 4-epimerase
MG_047	S-adenosylmethionine synthetase
MG_463	dimethyladenosine transferase
MG_360	ImpB/MucB/SamB family protein
MG_179	metal ion (cobalt) ABC transporter, ATP-binding protein, putative
MG_008	tRNA modification GTPase TrmE
MG_184	adenine-specific DNA modification methylase
MG_387	GTP-binding protein Era

Table 6. A list of 44 genes of *Mycoplasma genitaluim* genome that, when removed, provide the failure of genome informational laws. Table reports gene identifiers and their description provided in the *WholeCellKB* database (*http://www.wholecellkb.org/list/Mgenitalium/Gene*). The selection of the reported genes was done iteratively by making use of a greedy removal procedure. At each step, the gene is removed, together with its genomic context, that minimizes the *AF* value. Then, the procedure is applied iteratively to the reduced genome. Genes are reported in the same order they were selected. After 44 gene deletions, we obtained a sequence of length $487,137$, hosting 476 genes, for which the fourth law (see Equation 8 in the paper) does not hold. Further gene removals produce sequences where other laws fail (even if the removal do not minimize *AF*).
